# Evolution of breastfeeding indicators and early introduction of foods in Latin American and Caribbean countries in the decades of 1990, 2000 and 2010

**DOI:** 10.1186/s13006-022-00477-6

**Published:** 2022-04-22

**Authors:** Camila Abadia Rodrigues Meira, Gabriela Buccini, Catarina Machado Azeredo, Wolney Lisbôa Conde, Ana Elisa Madalena Rinaldi

**Affiliations:** 1 School of Medicine, Federal University of Uberlândia, Uberlândia, Brazil; 2 School of Public Health, University of Nevada, Las Vegas, USA; 3 School of Public Health, University of São Paulo, São Paulo, Brazil

**Keywords:** Breastfeeding, Weaning, Food consumption, Latin America

## Abstract

**Background:**

Early introduction of liquid/solid food before 6 months of age is one of the major barriers to exclusive breastfeeding. Our objective was to analyze the evolution of infant feeding practices for infants under 6 months of age in Latin American and Caribbean countries in the decades of 1990, 2000 and 2010.

**Method:**

Cross-sectional time series study with data from Demographic and Health Surveys carried out between 1990 and 2017 in six Latin America and Caribbean countries: Bolivia (1994 to 2008), Colombia (1995 to 2010), Dominican Republic (1996 to 2013), Guatemala (1995 to 2015), Haiti (1994/1995 to 2016/2017), Peru (1996 to 2018). Pooled sample comprised of 22,545 infants under the age of 6 months. Surveys were grouped in three decades: 1990s for surveys from 1990 to 1999, 2000s for surveys from 2000 to 2009, and 2010s for surveys from 2010 to 2017. Exclusive breastfeeding (EBF), predominant breastfeeding (PBF), mixed breastfeeding (mixed BF), supplemented breastfeeding (supplemented BF) and non-breastfeeding (non-BF), and individual foods (water, liquids, milk, infant formula, semi-solid/solid) were analyzed. Prevalence of breastfeeding and food indicators were calculated in pooled sample, according to the infant monthly age groups, decade of survey and residence area(rural/urban).

**Results:**

Between 1990s and 2010s, there was an increase in the exclusive breastfeeding prevalence (1990s = 38.1%, 2010s = 46.6%) and a reduction in the PBF prevalence (1990s = 51.7%, 2010 s = 43.1%). There was a decrease in the liquids (1990s = 40.7%, 2010s = 15.8%) and milk prevalence (1990s = 20.4%, 2010s = 8.3%) and an increase in water (1990s = 32.3%, 2010s = 37.6%), and infant formula (1990s = 16.6%, 2010s = 25.5%) prevalence. All breastfeeding indicators, except exclusive breastfeeding, progressively increased according to the monthly age group in three decades, and EBF prevalence sharply decreased from 2 to 3 months of age in all decades. Exclusive breastfeeding prevalence was higher in rural area in the three decades (1990s _rural_ = 43.8%, 1990s _urban_ = 32.4%, 2010s _rural_ = 51.1%, 2010s _urban_ = 42.4%) and infant formula prevalence was higher in urban area (1990s _rural_ = 8.6%, 1990s _urban_ = 24.6%, 2010s _rural_ = 15.9%, 2010s _urban_ = 34.1%).

**Conclusions:**

In the last three decades, in all age groups, there was an increase in exclusive breastfeeding prevalence, as well as a significant reduction in liquids and milk. In the rural area, EBF prevalence remains higher than in urban. Increased water and infant formula feeding are the main barriers to achieving the Global Nutrition Target 2025 for exclusive breastfeeding.

**Supplementary Information:**

The online version contains supplementary material available at 10.1186/s13006-022-00477-6.

## Background

Strong evidence supports the benefits of exclusive breastfeeding (EBF) for both children and mothers [1, 2]. The short-term benefits of EBF for children are mainly the prevention of diarrhea and respiratory diseases, identified as the main causes of infant mortality and the long-term benefits are the prevention of childhood obesity, chronic diseases in adult life and reduced risk of diabetes mellitus [1]. The benefits of breastfeeding for women are protection against breast cancer, ovarian cancer and type 2 diabetes, as well as improving birth spacing [2].

In 2012, the World Health Organization (WHO) adopted a resolution on maternal and child nutrition that included six Global Nutrition Targets. One of them sets a goal for countries to reach a 50% prevalence of EBF by 2025 [3]. However, a recent analysis pointed out that about 163 countries are not on course to meet the EBF target by 2025 [3]. In fact, in the region of Latin America and the Caribbean, the exclusive breastfeeding rate increased slightly from 35% in 2005 to 38% in 2018, but at this rate of annual growth it would take more than 40 years for the global EBF target to be reached [3]. Thus, it is critical to understand the barriers for EBF in Latin American and Caribbean countries [4].

The early introduction of solid food before 6 months of age is one of the multiple barriers for exclusive breastfeeding [5]. Other barriers include factors related to (i) the organization of support services for the management of breastfeeding (BF), such as the lack of preparation of health professional teams, (ii) political factors such as the absence or short duration of maternity leave [6, 7], (iii) cultural factors such as the perception of insufficient milk production by the mother and the use of a pacifier [8, 9], (iv) the partial implementation and weak monitoring of the International Code of Marketing of Breast-milk Substitutes (WHO Code) which may contribute to the unrestricted promotion of infant formula [6].

Although studies on the trend of breastfeeding in Latin America describe an increase in its duration until the end of the 2000s [10, 11], there are no investigations on the types of foods that are offered in addition to or as a substitute for breast milk and how much each food contributed to the interruption of EBF over three decades (1990s, 2000s and 2010s). This investigation can indicate what the main foods being offered to infants that prevent the fulfillment of the goal proposed by the WHO. Thus, the objective of this study was to analyze the evolution of infant feeding practices for children under 6 months of age in Latin American and Caribbean countries in the decades of 1990, 2000 and 2010.

## Methods

### Study design and data source

This is a cross-sectional time-series study carried out using data from the Demographic and Health Surveys (DHS) Program conducted in the decades of 1990, 2000 and 2010. The DHS Surveys are household-based surveys comparable across countries and within countries over different time periods with national representation that provide a broad set of data and indicators for monitoring and evaluating impact on demography, health and nutrition, and use these data for policy development, planning of monitoring, and evaluation programs. All data are available on the DHS Program website [12]. All surveys came from the DHS, with the exception of the last survey from Peru in 2018 included in the study through the *Encuesta Demográfica y de Salud Familiar* (ENDES) of the National Institute of Statistics and Informatics (INEI) of Peru [13]. Both variables and sampling strategy are similar in ENDES DHS surveys. The studies used were previously approved by ethics committees in each country, and the consent form was presented before the interview and signed by the respondents.

### Selected countries

The inclusion criteria for the countries were the existence of at least two DHS surveys between the 1990s and 2010s and available variables on foods consumed by the child the day before the survey (see Additional file 1). Based on these criteria, six Latin American countries and a total of 25 databases were included (Table 1): Bolivia (4 databases), Colombia (4 databases), Dominican Republic (4 databases), Guatemala (3 databases), Haiti (5 databases) and Peru (5 databases).

**Table 1:** Description of sample characteristics and characterization of pro-breastfeeding actions

Country Year	Study phase	Sample size	Legal status of the Code	Maternity leave	Births in BFHI (%)	Districts Implementing Community Programs (%)	Primary Healthcare Facilities with Individual IYCF Counselling (%)	Compliance with C183 and R191	Donor Funding (USD) Per Live Birth	Most Recent WBTi Breastfeeding Program Assessment
**Bolívia**										
1994	DHS III	615	Moderately aligned	Implemented	9.8	100	100	Does not meet	1.70	2017
1998	DHS III	743								
2003	DHS IV	906								
2008	DHS V	883								
**Colombia**										
1995	DHS III	536	Moderately aligned	Implemented	No information available	100	No information available	Meets recommendations	0.04	2017
2000	DHS IV	503								
2005	DHS V	1545								
2010	DHS VI	1703								
**Guatemala**										
1995	DHS III	1015	Moderately aligned	Implemented	1.2	100	100	Does not meet	0.66	2015
1998/1999	DHS IV	514								
2014/2015	DHS VII	1264								
**Haiti**										
1994/1995	DHS III	341	No information available	Implemented	9.5	100	100	Does not meet	9.40	WBTi was not assessed
2000	DHS IV	627								
2005/2006	DHS V	648								
2012	DHS VI	789								
2016/2017	DHS VII	695								
**Peru**										
1996	DHS III	1586	Moderately aligned	Implemented	3.0	25.9	No information available	Does not meet	3.23	2017
2000	DHS IV	1235								
2007/2008	DHS V	1675								
2012	DHS VI	892								
2018	ENDES-INEI	1912								
**Dominican Republic**										
1996	DHS III	419	Moderately aligned	Implemented	12.3	36.7	No information available	Does not meet	0.24	2017
1999	DHS IV	53								
2002	DHS IV	1100								
2013	DHS VI	346								

BFHI: Baby Friendly Hospitals and Maternities, Maternity Protection Convention Number 183 (C183) and Maternity Protection Recommendations Number 191 (R191) WBTi: World Breastfeeding Trends Initiative

Next, the enabling environment for breastfeeding in each country was synthesized based on documents from the United Nations Children’s Fund (UNICEF) and the International Labor Organization (ILO) (Table 1). In summary, Haiti was the only country where the International Code of Marketing of Breast-milk Substitutes has not been implemented. The presence of maternity leave was verified in all countries. The percentage of hospitals accredited as child-friendly ranged from 1.2% in Guatemala to 12.3% in the Dominican Republic and the percentage of Districts Implementing Community Programs ranged from 36.7% in the Dominican Republic to 100% in all other countries, except for Peru, which did not have such a pro-breastfeeding action. The percentage of Primary Healthcare Facilities with Individual Infant and young child feeding (IYCF) Counselling was 100% in three out of six countries. Only Colombia did not present compliance with ILO Maternity Protection Convention Number 183 (C183) and Maternity Protection Recommendations Number 191 (R191). Donor Funding (in USD) per live birth ranges from $0.04 in Colombia to $9.40 in Haiti. The Most Recent EBF Report took place between 2014 and 2018, and the most recent assessment tool from World Breastfeeding Trends Initiative (WBTi) took place between 2015 and 2017, except for Haiti, which did not carry out any assessments of the tool (Table 1).

### Analytical sampling and study population

The sampling procedure of all surveys selected for this study were considered standard sampling of DHS surveys [14]. All DHS and the ENDES surveys are household-based with complex sampling stratified in two-stages, with clusters (primary sampling units) being selected in the first stage, drawn from the most recent available Census files, and households (secondary sampling units) in the second stage, selected from an updated list of households [14, 15]. Samples from countries are representative at national, residence (rural/urban) and regional levels [14, 15].

Our study population consisted of infants under 6 months of age who were alive at the time of the interview and who lived with the respondent (see Additional file 2). Based on the established criteria, the total analytical sample consisted of 22,545 infants under 6 months of age. The description of the sample size according to year and phase of the study are described in Table 1. Exclusion criteria were dead infants and infants who did not live with the respondent. The percentage of excluded infants ranged from 1.3% in Colombia to 4.6% in Haiti.

### Breastfeeding and food indicators

Breastfeeding indicators analyzed were: Exclusive breastfeeding (EBF), predominant breastfeeding (PBF), mixed breastfeeding (mixed BF), and supplemented breastfeeding (supplemented BF). Infants who were not receiving breast milk were grouped under the heading non-breastfeeding (non-BF) (see Additional file 3). The description of breastfeeding indicators and food consumption is described in supplementary material (see Additional file 3).

Exclusive breastfeeding is defined as feeding breast milk only (numerator: infants under 6 months who are in EBF in the previous 24 h/denominator: infants aged 0–5 months), PBF as the provision of breast milk and other water-based liquids (numerator: infants aged 0–5 months who received breast milk and other water-based liquids in the previous 24 h/denominator: infants aged 0–5 months), mixed BF is the provision of breast milk supplemented with other types of milk and infant formula (numerator: infants aged 0–5 months who received breast milk, milk and formula in the previous 24 h/denominator: infants aged 0–5 months) and supplemented BF is defined as giving breast milk with semi-solid/solid foods (numerator: infants aged 0–5 months who received breast milk and solid foods/semi-solids in the previous 24 h/denominator: infants aged 0–5 months). In non-BF, the infant received any food other than breast milk in the previous 24 h.

Breastfeeding indicators adopted in this study were defined following the WHO recommendation [16–18]. All breastfeeding indicators were categorized as dichotomous variables (no/yes).

Food indicators analyzed were: water, liquids (teas, juices, soft drinks and other water-based liquids), milk, formula and semi-solid/solid foods. Food indicators collected were different across years of surveys and countries, ranging from 12 to 28 variables (see Additional file 1: Table S1). All food indicators referred to consumption on the day before the interview (i.e., in the previous 24 h) and were categorized as dichotomous variables (no/yes) (see Additional file 1: Table S1). In Bolivia in 2003, Colombia in 2000 and 2005, the Dominican Republic in 2002, Haiti in 2000, and Peru in 2000, the food variables were available as the number of times a day the food was consumed (0 to 7 times in the last 24 h) and were also configured as “no/yes”, with the objective of making the comparison between the studies conducted in different years compatible. Consumption equal to or greater than one was considered as “yes”. Missing data and the category “do not know” in the questions about food were considered as “not consumed”, as recommended by the WHO [16].

### Data analysis

All analyzes were conducted for the pooled sample (i.e., a merged sample with the six countries in Latin America and the Caribbean) and for the country sample (see Additional file 2: Table S2). For the analysis of the decades, the following argument was considered: surveys from 1990 to 1999 were grouped as 1990s, surveys from 2000 to 2009 as 2000s, and surveys from 2010 to 2017 as 2010s. All analyzes were performed using STATA SE® version 14.0.

First, the prevalence rates of breastfeeding and food indicators were calculated separately for each country and year of the survey considering the sample design and the weighting factor (country sample). Second, the samples from each country were grouped, and the pooled prevalence and confidence interval were calculated. Due to complex sampling, all analyzes were weighted by the effect of the sampling design of each country and each year of the survey (pooled sample). Third, pooled prevalence and confidence interval of breastfeeding and food indicators by monthly age group per decade were calculated for the pooled sample. Also, the pooled prevalence and 95% confidence interval of breastfeeding and food indicators were estimated by residence area (urban/rural) and survey years. The statistically significant differences of breastfeeding and food indicators between decades and age range were analyzed by confidence interval. For each country, we estimated the prevalence and confidence interval for all breastfeeding and food indicators. Within each country, linear regression weighted by variance was used for trend analysis, whose beta coefficient represented the annual average change between survey years for the breastfeeding and food indicators (see Additional files, Figs. S2, S3, S4, S5, S6 and S7). For each country, breastfeeding indicators were described by monthly age and decade using area charts (see Additional files, Figs. S8, S9, S10, S11, S12 and S13).

## Results

In the pooled analysis, we observed a trend towards an increase in EBF prevalence between 1990s and 2000s and 1990s and 2010s, however without difference between 2000s and 2010s decades. On the other hand, there was a progressive decrease in PBF prevalence in this period, especially from 2000s decade (Table 2). Considering the prevalence of food consumption per decade, we observed that liquids and milk showed a significant and progressive reduction in the prevalence in the three decades. We observed an increase in water and formula consumption between 1990s and 2000s decades and a maintenance of prevalence between 2000s and 2010s (Table 2). At the country level, there was an increase in EBF in all countries except for the Dominican Republic, a reduction in PBF in all countries except for the Dominican Republic, a reduction in mixed BF in three countries and an increase in the Dominican Republic, and a reduction in supplemented BF in three out of six countries (see Additional files, Figs. S2, S3, S4, S5, S6 and S7).

**Table 2:** Prevalence and confidence interval (95% CI) of breastfeeding indicators and food indicators according to decades. DHS, 1990s, 2000s, 2010s. ENDES, 2018

	Decades		
	1990	2000	2010
Breastfeeding indicators	**% (95%CI)**		
EBF	38.1(36.3,39.9)	45.3(43.7,46.9)	46.6(44.7,48.4)
PBF	51.7(48.9,53.5)	47.6(45.9,49.2)	43.1(41.3,44.9)
Mixed BF	34.5(32.8,36.2)	37.7(36.2,39.3)	32.7(31.1,34.4)
Supplemented BF	23.7(22.2,25.3)	22.3(20.9,23.6)	24.5(23.0,26.1)
Non-BF	5.4(4.7,6.2)	6.4(5.7,7.2)	6.7(5.8,7.7)
Food indicators	**% (95%CI)**		
Water	32.3(30.7,34.0)	38.5(36.9,40.2)	37.6(35.9,39.4)
Liquids	40.7(38.9,42.3)	22.3(21.2,23.5)	15.8(14.6,17.1)
Milk	20.4(19.1,21.8)	13.0(12.1,14.1)	8.3(7.4,9.4)
Formula	16.6(15.3,17.9)	25.3(23.9,26.7)	25.5(23.9,27.0)
Semi-solids/solids	23.7(22.2,25.3)	22.3(20.9,23.6)	24.5(23.0,26.1)

*BF* breastfeeding, *EBF* exclusive breastfeeding, *PBF* predominant breastfeeding

For the prevalence of breastfeeding indicators according to the children monthly age group (0–5 months), we noted a dose-response with progressive reduction in the prevalence of EBF as children age group increased. There was an increase of EBF prevalence for 0, 1- and 2-months age groups between 1990s and 2010s. For 3 months age group or older, the prevalence of EBF was similar across the three decades (Table 3). The prevalence of PBF increased between 0- and 2-months age group across decades and the prevalence was similar from 3 to 5 age group. There was a decrease of PBF prevalence between 1990s and 2010s for 0 to 3 months age group infants (Table 3). Mixed and supplemented BF and non-BF prevalence increased according to age group and the prevalence were similar between decades.

**Table 3:** Prevalence and confidence interval (95% CI) of breastfeeding indicators according to monthly age group and decade. DHS, 1990s, 2000s, 2010s. ENDES, 2018

Breastfeeding indicators	Decades	Age group (month)					
		0	1	2	3	4	5
		Prevalence (95% CI)					
**EBF**	1990	58.5(54.5,62.4)	50.1(45.7,54.5)	38.5(34.6,42.5)	37.5(33.7,41.5)	29.4(25.9,33.3)	22.1(19.2,25.4)
	2000	65.5(61.9,68.8)	58.6(54.7,62.4)	48.7(45.4,52.0)	40.9(37.4,44.7)	36.2(33.2,39.2)	23.9(21.3,26.9)
	2010	67.6(63.0,71.8)	63.2(58.5,67.6)	51.4(47.6,55.2)	45.4(41.3,49.6)	33.9(30.5,37.6)	22.2(19.1,25.6)
**PBF**	1990	29.6(26.0,33.5)	39.0(34.8,43.3)	50.2(45.9,54.4)	52.7(48.6,56.7)	62.7(58.7,66.6)	68.2(64.6,71.5)
	2000	26.9(23.7,30.3)	34.5(30.7,38.5)	41.2(37.7,44.7)	51.5(47.7,55.3)	57.6(54.3,60.7)	69.4(66.1,72.5)
	2010	21.5(17.9,25.5)	27.5(23.4,32.0)	37.3(33.7,40.9)	45.1(40.9,49.2)	55.0(51.1,58.8)	68.0(64.2,71.6)
**Mixed BF**	1990	22.0(18.9,25.4)	30.6(26.7,34.9)	34.7(31.2,38.3)	35.5(31.9,39.2)	38.2(34.5,41.9)	42.3(38.6,46.1)
	2000	27.3(24.1,30.9)	29.9(26.3,33.8)	38.5(35.2,41.9)	40.7(37.1,44.5)	41.8(38.7,45.1)	44.9(41.6,48.3)
	2010	24.8(20.9,29.2)	23.9(20.3,27.9)	33.2(29.7,36.9)	32.4(28.7,36.4)	36.8(33.2,40.7)	42.7(38.7,46.8)
**Supplemented BF**	1990	3.8(2.5,5.9)	6.3(4.6,8.6)	12.3(10.1,14.8)	23.2(20.1,26.7)	34.7(31.2,38.4)	54.6(50.7,58.4)
	2000	2.9(1.9,4.3)	5.5(3.7,8.1)	9.8(8.2,11.7)	20.5(17.6,23.7)	35.1(32.0,38.4)	55.4(51.9,58.7)
	2010	4.9(3.4,7.1)	8.3(6.1,11.2)	14.3(11.9,16.9)	22.4(18.9,26.3)	35.9(32.4,39.6)	58.9(54.8,62.8)
**Non-BF**	1990	1.3(0.7,2.4)	2.5(1.4,4.2)	4.2(3.0,5.7)	5.7(4.1,7.8)	7.8(5.9,10.4)	9.6(7.7,11.9)
	2000	1.6(0.9,2.6)	2.1(1.2,3.9)	5.5(4.1,7.4)	6.8(5.2,8.8)	9.9(8.0,12.2)	11.6(9.7,13.9)
	2010	3.7(2.3,5.9)	2.7(1.7,4.5)	6.2(4.5,8.3)	5.6(3.9,7.9)	8.1(6.0,10.7)	13.2(10.4,16.5)

*BF* breastfeeding, *EBF* exclusive breastfeeding, *PBF* predominant breastfeeding

At the country level, from 0-to-3-month age group, EBF, followed by PBF and mixed BF were the most prevalent indicators. On the other hand, from the 2nd or 3rd month onwards, the EBF prevalence reduced progressively, accompanied by an increase in the introduction of other milk with breastfeeding (mixed BF) or semi-solid and solid foods (complemented BF). For all countries, except in the Dominican Republic, the prevalence of EBF increased between 1990s to 2010s decades. From 0 to 3 months, the prevalence of supplemented BF decreased, and from the 3rd month to the 5th month increased. Non-BF indicator increased as the infant’s age increased (see Additional files, Figs. S8, S9, S10, S11, S12 and S13).

When analyzing the food indicators separately, we found an increase in consumption with increasing child’s age across decades (Table 4). The prevalence of water consumption increased especially between the two first months of age, and from 2 months age group the prevalence was similar for all decades. There was a significant reduction in the prevalence of liquids and milk, progressively between the 1990s and 2010s, in all age groups. The prevalence of infant formula increased between decades, especially in the 2000s, and the prevalence was similar across all age groups within each decade (Table 4). We identified a progressive increase in semi-solids/solids, especially between the 3rd and 4th month of age, but the prevalence for all age groups was similar across decades. At the country level, we found a significant reduction in water in Haiti (− 1.9 percentage points) and a less pronounced reduction in Bolivia and Colombia. In all countries, except for the Dominican Republic, we observed a significant reduction in liquids and milk. There was an increase in formula consumption in 50% of the countries and a reduction in semi-solids/solids in four out of six countries (see Additional files, Figs. S2, S3, S4, S5, S6 and S7).

**Table 4:** Prevalence and confidence interval (95% CI) of food indicators according monthly age group and decade, DHS, 1990s, 2000s, 2010s. ENDES, 2018

Foods	Decades	Age group (month)					
		0	1	2	3	4	5
		Prevalence (95% CI)					
**Water**	1990	17.9(15.1,21.3)	23.1(19.8,26.7)	33.3(29.6,37.2)	34.8(31.0,38.7)	38.2(34.5,42.1)	41.3(37.6,44.9)
	2000	21.1(18.2,24.6)	28.6(25.0,32.5)	35.4(32.0,38.9)	43.8(40.0,47.7)	45.8(42.6,49.1)	53.0(49.5,56.4)
	2010	18.7(15.4,22.6)	23.3(19.6,27.4)	34.5(30.9,38.1)	42.1(38.0,46.2)	47.5(43.7,51.4)	55.6(51.6,59.5)
**Liquids**	1990	21.6(18.3,25.2)	27.9(24.1,31.9)	36.6(32.8,40.4)	40.2(36.5,43.9)	52.4(48.3,56.4)	58.5(54.9,62.0)
	2000	7.9(6.3,10.1)	9.6(7.8,11.9)	13.3(11.3,15.5)	21.3(18.5,24.3)	32.1(29.3,35.1)	49.1(45.7,52.5)
	2010	5.5(3.8,8.1)	5.9(4.1,8.6)	9.4(7.6,11.7)	12.2(9.7,15.1)	21.9(18.9,25.4)	38.7(34.8,42.8)
**Milk**	1990	10.0(7.9,12.7)	13.1(10.5,16.4)	18.5(15.9,21.4)	19.7(16.9,22.9)	25.2(22.1,28.6)	31.9(28.6,35.50
	2000	5.3(4.0,7.0)	6.5(4.9,8.6)	10.5(8.7,12.6)	12.7(10.6,15.2)	18.9(16.5,21.5)	23.4(20.7,26.4)
	2010	4.0(2.5,6.4)	3.1(1.9,4.8)	7.4(5.7,9.7)	6.9(5.3,9.2)	10.9(8.8,13.5)	16.3(13.4,19.6)
**Formula**	1990	13.4(11.0,16.3)	19.2(15.8,23.1)	17.6(15.2,20.4)	18.6(15.7,21.8)	16.3(13.4,19.6)	14.3(11.9,17.0)
	2000	22.1(19.1,25.5)	23.3(19.9,26.9)	28.3(25.1,31.7)	28.6(25.3,32.2)	24.5(21.9,27.5)	23.7(20.7,26.8)
	2010	21.2(17.5,25.3)	21.2(17.8,25.1)	26.9(23.7,30.5)	26.7(23.1,30.6)	27.3(23.8,31.0)	27.9(24.3,31.7)
**Semi-solids/solids**	1990	3.8(2.5,5.9)	6.3(4.6,8.6)	12.3(10.1,14.8)	23.2(20.1,26.7)	34.7(31.2,38.4)	54.6(50.7,58.4)
	2000	2.9(1.9,4.3)	5.5(3.7,8.1)	9.8(8.2,11.7)	20.5(17.6,23.7)	35.1(32.0,38.4)	55.4(51.9,58.7)
	2010	4.9(3.4,7.1)	8.3(6.1,11.2)	14.3(11.9,16.9)	22.4(18.9,26.3)	35.9(32.4,39.6)	58.9(54.8,62.8)

According to the area of residence from 1990 to 2010, the prevalence of EBF was higher in infants living in rural areas across all decades (Table 5). There was an increase in the EBF prevalence in both rural and urban area between 1990s and 2000s and a maintenance between 2000s and 2010s. The prevalence of PBF was higher in rural area only the 1990s and there was a decrease between 1990s and 2000s. The prevalence of mixed BF and non-BF was higher in urban area in all decades and the prevalence was similar across decades. The prevalence of supplemented BF was similar in rural and urban areas and across decades. The prevalence of liquids and milks was higher in urban areas in the 1990s and decreased in both areas across decades. The prevalence of water and semi-solid/solid foods was similar in rural and urban areas. The prevalence of infant formula was higher in urban area in all decades (Table 5).

**Table 5:** Prevalence and confidence interval (95%CI) of breastfeeding and food indicators according to area of residence and decade. DHS, 1990s, 2000s, 2010s. ENDES, 2018

Indicators and foods	Decades					
	1990s		2000s		2010s	
	rural	urban	rural	urban	rural	urban
**Breastfeeding indicators**	**Prevalence (95% CI)**					
**EBF**	43.8(41.3,46.3)	32.4(29.9,35.0)	51.2(48.7,53.7)	41.0(38.9,43.1)	51.1(48.5,53.7)	42.4(39.9,44.9)
**PBF**	48.6(46.1,51.1)	54.8(52.2,57.3)	42.4(40.0,44.8)	45.8(43.8,47.8)	42.6(40.0,45.2)	43.5(41.1,45.9)
**Mixed BF**	24.4(22.4,26.6)	44.7(42.2,47.3)	24.4(22.7,26.3)	43.1(41.1,45.2)	22.5(20.5,24.6)	41.9(39.6,44.4)
**Supplemented BF**	21.9(19.9,24.0)	25.6(23.3,27.9)	21.7(19.8,23.7)	20.1(18.6,21.7)	26.1(23.9,28.4)	23.1(21.1,25.2)
**Non-BF**	3.2(2.5,4.0)	7.7(6.5,9.1)	3.5(2.8,4.3)	8.6(7.5,9.7)	3.9(3.0,4.9)	9.2(7.9,10.9)
**Foods**	**Prevalence (95% CI)**					
**Water**	32.3(29.9,34.8)	32.3(30.1,34.6)	34.9(32.5,37.5)	36.7(34.7,38.7)	38.2(35.8,40.8)	37.1(34.8,39.5)
**Liquids**	37.1(34.9,39.3)	44.3(41.8,46.7)	20.6(19.1,22.3)	23.6(22.0,25.2)	14.1(12.5,15.9)	17.4(15.6,19.3)
**Milk**	17.0(15.3,18.9)	23.9(21.9,25.9)	12.4(11.1,13.9)	13.5(12.2,14.9)	7.1(5.9,8.6)	9.4(7.1,10.9)
**Formula**	8.6(7.4,10.0)	24.6(22.5,26.8)	12.9(11.6,14.5)	31.4(29.4,33.4)	15.9(14.3,17.7)	34.1(31.8,36.6)
**Semi-solids/solids**	21.9(19.9,24.0)	25.6(23.3,27.9)	21.7(19.8,23.7)	20.1(18.6,21.7)	26.1(23.9,28.4)	23.1(21.1,25.2)

*BF* breastfeeding, *EBF* exclusive breastfeeding, *PBF* predominant breastfeeding

## Discussion

Our study is the first known to explore barriers to achieving the Global Nutrition Target 2025 for Exclusive Breastfeeding in Latin America and the Caribbean. Our results showed an increase in EBF prevalence and a reduction in PBF between 1990s and 2000s and these changes were sustained in 2010s decade. As expected, we found that breastfeeding and infant feeding indicators vary depending on the infant’s age. Between zero and 3 months, there was an increase in the prevalence of exclusive breastfeeding over the decades. Among the foods offered to infants, the significant reduction in the prevalence of milk and liquids in all age groups may have contributed, in part, to the increase in EBF among infants under 3 months of age. The increased prevalence of formulas and water may have contributed, in part, to the maintenance of supplemented BF, which remained virtually the same for all age groups, being less than 10% for those under the age of 2 months, reaching close to 50% at 5 months. Over the three decades, we observed a higher prevalence of EBF and a lower prevalence of formulas in rural areas. Thus, strategies to promote breastfeeding with a focus on reducing the early supply of water and formulas are potential methods for the Latin American and Caribbean region to reach the threshold of 50 and 70% of exclusive breastfeeding established by the Global Nutrition Target 2025 and 2030, respectively.

We observed an increase in the prevalence of exclusive breastfeeding over the decades, especially in the age range from birth to the second month of life. While these findings indicate that perhaps more infants have been exposed to EBF since birth, on the other hand, we found a high consumption of liquids, milk and especially water in the first month of life, possibly due to cultural issues, and the lack of guidance in breastfeeding after birth in hospitals. Although the Baby-Friendly Hospital Initiative (BFHI) has been implemented in all study countries, the numbers of births performed in hospitals with this initiative are still very low. Babies born in the BFHI are more likely to be breastfed upon hospital discharge and maintain EBF for up to 6 months [3, 19, 20]. This is because the BFHI favors the training of health professionals to support mothers and infants on the initial difficulties of breastfeeding, through adequate guidance and support in hospitals right after birth, which also helps to increase EBF rates [19]. In this sense, a higher percentage of BFHI in the countries studied may favor the practice of EBF, and reduce the early introduction of water, liquids and milk in the first months of life [3, 21].

From the second and third month on, there is a sharp drop in exclusive breastfeeding rates in all years of study and in all countries. The drop in EBF from the third month on seems to coincide with the duration of maternity leave in the countries studied. The duration of maternity leave in the countries studied varies from 12 to 14 weeks, that is, the end of maternity leave may partly explain the drop in EBF in this age group, and contribute to the early introduction of food before 6 months of age [22]. Evidence has shown that support in the workplace [23], such as flexible working hours and a suitable place to extract breast milk or breastfeed [24] can support the maintenance of exclusive breastfeeding among working mothers [2, 25]. On the other hand, the informal market in Latin America is very large and legal protection such as maternity leave is not available for those women, therefore measures to protect informal workers are necessary. An alternative to make maternity leave available to the women who are in the informal market in Latin America and the Caribbean is the transfer of maternity income [26]. Another potential explanation for the drop in exclusive breastfeeding from the third month of life onwards, it may be related to threats to the maternal self-efficacy in maintaining exclusive breastfeeding due to changes in the baby’s eating and crying behaviors, in the volume of the breast (due to regulation of baby’s demand), family beliefs about introduction of complementary food, pacifier use among others [8, 9, 27, 28].

Although we observed the increase in the prevalence of EBF between 1990s and 2010s decades, we also noticed its reduction according to the evolution of the monthly age groups between the 1990s and 2010s. These findings point to the need to invest in continued support for breastfeeding, through the implementation and scale up of individual and group infant and young child feeding counselling in community-based programs and Primary Healthcare Facilities [3]. Community programs play an important role in improving breastfeeding practices, precisely because they support women in maintaining and overcoming barriers during the breastfeeding period [3]. Evidence has shown that providing advice through qualified health professionals about the infant and young child feeding increases women’s knowledge, practice and confidence in breastfeeding [3]. Indeed, infant and young child feeding counseling interventions can increase global exclusive breastfeeding rates by 12–16% [29, 30]. In 2019, UNICEF indicated that globally 47% of countries have community programs that include infant and young child feeding counselling, which is far from proposed target of 70% by 2030 [3]. In order to achieve this goal, Latin America and the Caribbean must increase their capacity to finance breastfeeding programs [31].

In our study, we observed an increase in the prevalence rates of water between 1990s and 2010s decades according to the evolution of the monthly age groups, especially from the third month of life onwards. The consumption of water is present in a high percentage of infants under 6 months of age, and this consumption can place infants’ health at risk since there is the hypothesis of water contamination due to sanitation issues in Latin American and Caribbean countries [32]. Water seems to be one of the foods that most contributes to the early introduction of food in infants under 6 months of age, and this supply of water and other liquids such as teas and juices is strongly related to the maternal and family belief in the relief of colic and gases, and to quench the baby’s thirst [33].

According to residence area, we found a maintenance of higher prevalence of exclusive breastfeeding in a rural area, a lower prevalence of infant formula consumption and lower prevalence of non-breastfeeding infants in rural areas for all decades. There was an expressive reduction of liquids and milk in rural and urban areas. We hypothesized that an infant and their families living in rural areas can be more protected from the infant formula industry marketing and its commercialization, which is growing and is resilient to market downturns [6]. Previous studies showed lower prevalence of EBF in urban areas when compared to rural areas, corroborating the findings of our study [17, 34, 35]. In addition, the prevalence of infant formula has also increased in rural and urban areas over the decades.

In the same sense, we observed an increase in the prevalence of infant formulas over the decades and in all age groups of infants. The supply of milk other than breast milk is also related to the maternal belief of weak milk, and the maternal expectation of offering more energy and nutrients to the baby [36]. Additionally, problems related to the breasts in the immediate postpartum period may also influence the supply of formula or food supplement soon after birth [19, 37]. Evidence points to formula as one of the foods that most contributes to the early introduction of food before 6 months of life [19, 37]. On the other hand, consumption of infant formula is better for the infant’s health when compared to consumption of cow’s milk, other foods, and liquids before 6 months of age, but breast milk is the best food for the baby when compared to infant formula [18]. Some authors have attributed this change in dietary patterns, that is, the increase in the supply of formulas, and the decrease in the supply of other milk found in our study, to the improvement in the socioeconomic situation of Latin American and Caribbean countries with increased access to and sales of infant formulas [38, 39].

Although all countries in our study, except for Haiti, adopt all components of the International Code of Marketing of Breast-milk Substitutes (The Code), we have not identified public data on inspection and monitoring in the literature. In this context, strengthening the Code becomes even more critical in conjunction with other strategies to promote, protect, and support breastfeeding that can contribute to reducing the use of formulas [3, 40].

Our study has some limitations that need to be considered when interpreting the results. The first limitation is related to differences in the number of food indicators according to the year of study. The systematization of indicators by WHO in 2008 for Latin America and the Caribbean may have served as a guide for these countries to increase the number of food variables in the surveys. It is possible that this increase could make the frequency of EBF lower in the recent years compared to the previous years, as it gives the mother more food options to remember. However, we found an increase in exclusive breastfeeding over time, suggesting that this potential misclassification was not relevant. There was no data in DHS and ENDES about the age of food introduction, therefore, we could only estimate if infant consumed or not. Another limitation was the lack of more recent surveys for Bolivia and Colombia, as the most recent surveys for those countries in our dataset were conducted over 10 years ago. Although a DHS survey is available for Colombia in 2015, it does not have infant feeding data and therefore was not included in our dataset. We checked the UNICEF website [41] and searched the country websites, and found more recent surveys for Bolivia (2016), but there is no recent data on infant feeding. We also highlight as a limitation, the lack of data for some Latin American countries that have large populations of children under 2 years old, such as Brazil and Mexico. These countries represent important markets for infant formula companies.

Despite these limitations, the strengths of our study are the use of nationally representative surveys, analysis of six Latin American countries over a long period of time, and analysis by monthly age group of what was being offered to infants who were not exclusively breastfed, to understand if the type of food that interferes with breastfeeding has changed over time. Thus, our analyzes are important to support health professionals and especially health managers to understand the situation of infant feeding in the first 6 months of life in Latin America, as we observe that breast milk is being supplemented or replaced by infant formulas.

## Conclusions

Our study explored which foods could hinder the practice of exclusive breastfeeding between the 1990s, 2000s and 2010s. In the period from the 1990s to 2010s, we observed an increase in exclusive breastfeeding prevalence, especially in the first 3 months of the infant’s life. The consumption of liquids and milk decreased progressively across the three decades and age groups and can contribute to explain the increase in exclusive breastfeeding. The increase in prevalence of infant formula use, especially in urban area, together with sustained prevalence of early introduction of water may represent our major challenge to increase exclusive breastfeeding prevalence. Strategies to reduce the early introduction of water and infant formula are critical for Latin America and the Caribbean to reach the 50% exclusive breastfeeding target established by the Global Nutrition Target 2025.

## Supplementary Information

**Additional file 1: Table S1.** Description of food indicators number available in each survey questionnaire according to survey year and country. DHS, 1990 to 2017. ENDES, 2018.

**Additional file 2: Figure S1.** Flowchart of Demographic and Health Survey (DHS) selection from Latin American and Caribbean countries. DHS, 1990s, 2000s and 2010s. ENDES, 2018.

**Additional file 3: Table S2.** Description of infant feeding indicators configuration for all surveys year and countries. DHS, 1990–2017. ENDES, 2018.

**Additional file 4: Figure S2.** Prevalence of the breastfeeding and food indicators and average annual changes for indicators for children under six months of age in Bolívia according to the year of the survey. DHS, 1990–2008.

**Additional file 5: Figure S3.** Prevalence of the breastfeeding and food indicators and average annual changes for indicators for children under six months of age in Colombia according to the year of the survey. DHS, 1990–2010.

**Additional file 6: Figure S4.** Prevalence of the breastfeeding and food indicators and average annual changes for indicators for children under six months of age in Dominican Republic according to the year of the survey. DHS, 1990–2013.

**Additional file 7: Figure S5.** Prevalence of the breastfeeding and food indicators and average annual changes for indicators for children under six months of age in Guatemala according to the year of the survey. DHS, 1990–2014.

**Additional file 8: Figure S6.** Prevalence of the breastfeeding and food indicators and average annual changes for indicators for children under six months of age in Haiti according to the year of the survey. DHS, 1990–2016.

**Additional file 9: Figure S7.** Prevalence of the breastfeeding and food indicators and average annual changes for indicators for children under six months of age in Peru according to the year of the survey. DHS, 1990–2018. ENDES, 2018.

**Additional file 10: Figure S8.** Prevalence of breastfeeding indicators for infants under six months of age from Bolivia by survey year and monthly age group. DHS, 1994–2008.

**Additional file 11: Figure S9.** Prevalence of breastfeeding indicators for infants under six months of age from Colombia by survey year and monthly age group. DHS, 1995–2010.

**Additional file 12: Figure S10.** Prevalence of breastfeeding indicators for infants under six months of age from Dominican Republic by survey year and monthly age group, DHS, 1996–2013.

**Additional file 13: Figure S11.** Prevalence of breastfeeding indicators for infants under six months of age from Guatemala by survey year and monthly age group DHS, 1995–2015.

**Additional file 14: Figure S12.** Prevalence of breastfeeding indicators for infants under six months of age from Haiti by survey year and monthly age group. DHS, 1994–2017.

**Additional file 15: Figure S13.** Prevalence of breastfeeding indicators for infants under six months of age from Peru by survey year and monthly age group. DHS, 1996–2018. ENDES, 2018.

## Data Availability

The dataset is freely available for download at: https://dhsprogram.com/Countries/ http://iinei.inei.gob.pe/microdatos/

## Abbreviations

EBF: Exclusive Breastfeeding
PBF: Predominant Breastfeeding
Mixed BF: Mixed Breastfeeding
Supplemented BF: Supplemented Breastfeeding
Non-BF: Non-breastfeeding
