# Supplementary material for: Evolution of breastfeeding indicators and early introduction of foods in Latin American and Caribbean countries in the decades of 1990, 2000 and 2010
Source: Int Breastfeed J. 2022 Apr 22;17:32. doi: 10.1186/s13006-022-00477-6 (PMC9034574; doi:10.1186/s13006-022-00477-6)
Supplement: Supplementary file 1 — Additional file 1: Table S1. Description of food indicators number available in each survey questionnaire according to survey year and country. DHS, 1990 to 2017. ENDES, 2018. [file 13006_2022_477_MOESM1_ESM.docx]

**Table S1.** Description of food variables number available in each survey questionnaire according to survey year and country. DHS, 1990 to 2017. ENDES, 2018.

| **DHS-Year** | **Liquids*** | **Milk**** | **Semisolids/**  **solids** | **Total** |
| --- | --- | --- | --- | --- |
| **Bolivia** | | | | |
| DHS III-1994 | 5 | 3 | 5 | 13 |
| DHS III-1998 | 5 | 3 | 5 | 13 |
| DHS IV-2003 | 3 | 2 | 10 | 15 |
| DHS V-2008 | 6 | 2 | 16 | 24 |
| **Colombia** | | | | |
| DHS III-1995 | 5 | 3 | 5 | 13 |
| DHS IV-2000 | 3 | 2 | 9 | 14 |
| DHS V-2005 | 4 | 2 | 15 | 21 |
| DHS VI-2010 | 3 | 2 | 23 | 28 |
| **Dominican Republic** | | | | |
| DHS III-1996 | 6 | 3 | 6 | 15 |
| DHS IV-1999 | 3 | 2 | 7 | 12 |
| DHS IV-2002 | 3 | 2 | 10 | 15 |
| DHS VI-2013 | 3 | 2 | 16 | 21 |
| **Guatemala** | | | | |
| DHS III-1995 | 5 | 3 | 5 | 13 |
| DHS IV-1998/1999 | 5 | 3 | 5 | 13 |
| DHS VII-2014/2015 | 3 | 2 | 23 | 27 |
| **Haiti** | | | | |
| DHS III-1994/1995 | 6 | 3 | 3 | 12 |
| DHS IV-2000 | 3 | 2 | 10 | 15 |
| DHS V-2005/2006 | 5 | 2 | 17 | 24 |
| DHS VI-2012 | 3 | 2 | 17 | 22 |
| DHS VII-2016/2017 | 3 | 2 | 16 | 21 |
| **Peru** | | | | |
| DHS III-1996 | 5 | 4 | 5 | 14 |
| DHS IV-2000 | 3 | 2 | 11 | 16 |
| DHS V-2007/2008 | 4 | 2 | 16 | 22 |
| DHS VI-2012 | 4 | 3 | 16 | 23 |
| INEI-2018 | 4 | 3 | 16 | 23 |

*Liquids: water, sugar water, juice, fruit juice, herbal tea, other liquids, liquid, carbonated, soda, coffee, tea, other country-specific liquids, rice water, water with sugar or tea or coffee or soda or soup.

**Milk: infant formula, fresh milk, powdered milk, evaporated milk.
