# Supplementary material for: Evolution of breastfeeding indicators and early introduction of foods in Latin American and Caribbean countries in the decades of 1990, 2000 and 2010
Source: Int Breastfeed J. 2022 Apr 22;17:32. doi: 10.1186/s13006-022-00477-6 (PMC9034574; doi:10.1186/s13006-022-00477-6)
Supplement: Supplementary file 2 — Additional file 2: Figure S1. Flowchart of Demographic and Health Survey (DHS) selection from Latin American and Caribbean countries. DHS, 1990s, 2000s and 2010s. ENDES, 2018. [file 13006_2022_477_MOESM2_ESM.docx]

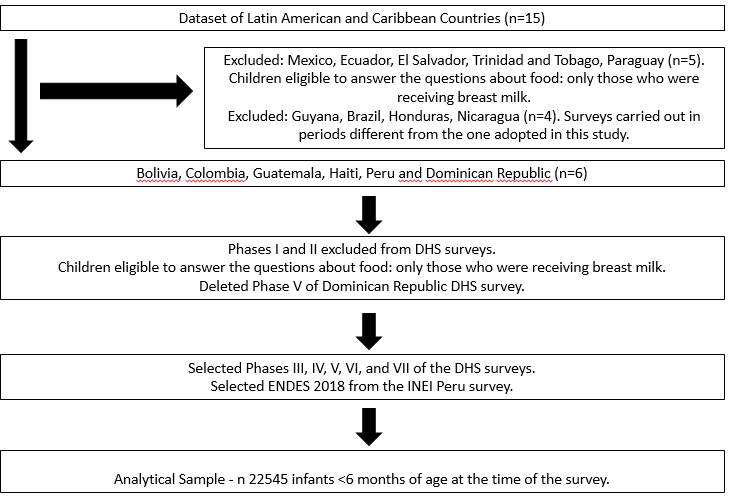


**Figure S1.** Flowchart of Demographic and Health Survey (DHS) selection from Latin American and Caribbean countries. DHS, 1990s, 2000s and 2010s. ENDES, 2018.
