# Supplementary material for: Evolution of breastfeeding indicators and early introduction of foods in Latin American and Caribbean countries in the decades of 1990, 2000 and 2010
Source: Int Breastfeed J. 2022 Apr 22;17:32. doi: 10.1186/s13006-022-00477-6 (PMC9034574; doi:10.1186/s13006-022-00477-6)
Supplement: Supplementary file 3 — Additional file 3: Table S2. Description of infant feeding indicators configuration for all surveys year and countries. DHS, 1990–2017. ENDES, 2018. [file 13006_2022_477_MOESM3_ESM.docx]

**Table S2.** Description of infant feeding variables configuration for all surveys year and countries. DHS, 1990-2017. ENDES, 2018.

| **Configuration of infant feeding indicators** | |
| --- | --- |
| Step 1: Configuration of variables related to power | -All food supply variables in the last 24 hours were set as 0=no and 1=yes (binary).  -For missing data, we replace for “0” (not offered”), as recommended by WHO (2008). |
| Step 2: EBF variable creation | -EBF variable created from the food variables configured in the previous step (all variables were categorized as 0 (no) or 1(yes)).  - Infants who did not consume foods were categorized as “exclusive breastfeeding”(1) and infants who consume any food were categorized as “non exclusivve breasfeding” (0). |
| Step 3: creation of variables: PBF, mixed BF, Supplemented BF and non-BF | - Variables created from the food variables configured in step 1.  - Infants who consumed liquid foods and breast milk were classified as “predominant breastfeeding”  - Infants who consumed milks and breast milk were classified as “mixed breastfeeding”  - Infants who consumed semi-solid/solid foods and breast milk were classified as “suplemmented breastfeeding”  -Infants who consumed other foods and did not receive breast milk were classified as “non-breastfed” |
