# Supplementary material for: Evolution of breastfeeding indicators and early introduction of foods in Latin American and Caribbean countries in the decades of 1990, 2000 and 2010
Source: Int Breastfeed J. 2022 Apr 22;17:32. doi: 10.1186/s13006-022-00477-6 (PMC9034574; doi:10.1186/s13006-022-00477-6)
Supplement: Supplementary file 7 — Additional file 7: Figure S5. Prevalence of the breastfeeding and food indicators and average annual changes for indicators for children under six months of age in Guatemala according to the year of the survey. DHS, 1990–2014. [file 13006_2022_477_MOESM7_ESM.docx]

**
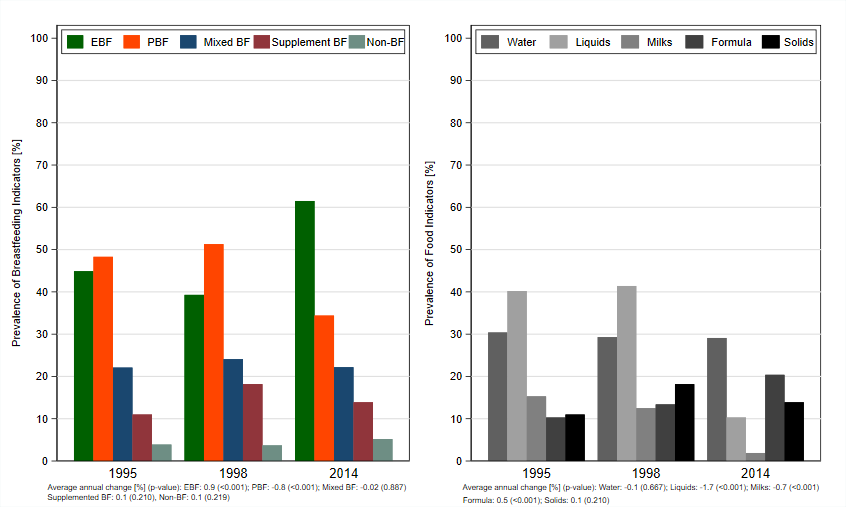
**

BF: breastfeeding; EBF: exclusive breastfeeding; PBF: predominant breastfeeding.

**Figure S5.** Prevalence of the breastfeeding and food indicators and average annual changes for indicators for children under six months of age in Guatemala according to the year of the survey. DHS, 1990-2014.
