# Supplementary material for: Evolution of breastfeeding indicators and early introduction of foods in Latin American and Caribbean countries in the decades of 1990, 2000 and 2010
Source: Int Breastfeed J. 2022 Apr 22;17:32. doi: 10.1186/s13006-022-00477-6 (PMC9034574; doi:10.1186/s13006-022-00477-6)
Supplement: Supplementary file 10 — Additional file 10: Figure S8. Prevalence of breastfeeding indicators for infants under six months of age from Bolivia by survey year and monthly age group. DHS, 1994–2008. [file 13006_2022_477_MOESM10_ESM.docx]

**
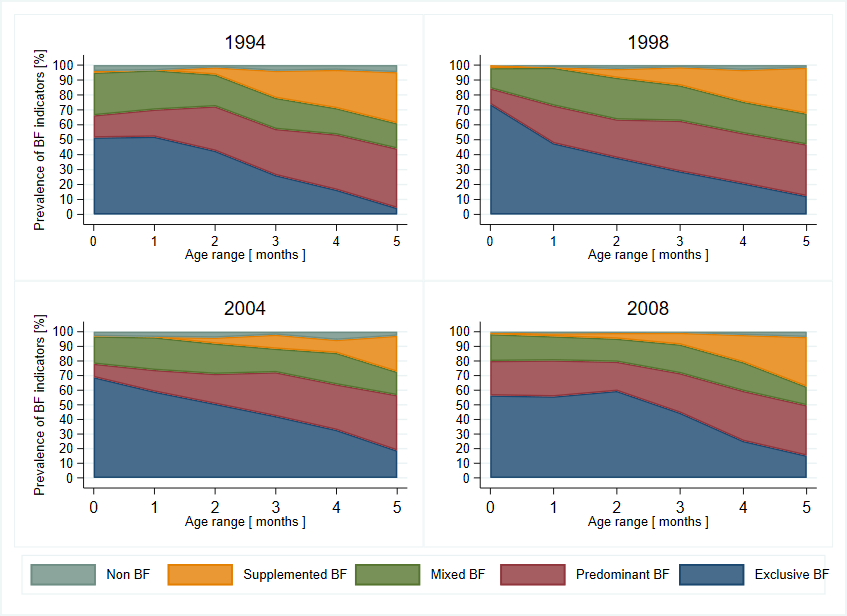
Figure S8.** Prevalence of breastfeeding indicators for infants under six months of age from Bolivia by survey year and montly age group. DHS, 1994-2008.
