# Supplementary material for: Evolution of breastfeeding indicators and early introduction of foods in Latin American and Caribbean countries in the decades of 1990, 2000 and 2010
Source: Int Breastfeed J. 2022 Apr 22;17:32. doi: 10.1186/s13006-022-00477-6 (PMC9034574; doi:10.1186/s13006-022-00477-6)
Supplement: Supplementary file 11 — Additional file 11: Figure S9. Prevalence of breastfeeding indicators for infants under six months of age from Colombia by survey year and monthly age group. DHS, 1995–2010. [file 13006_2022_477_MOESM11_ESM.docx]

**
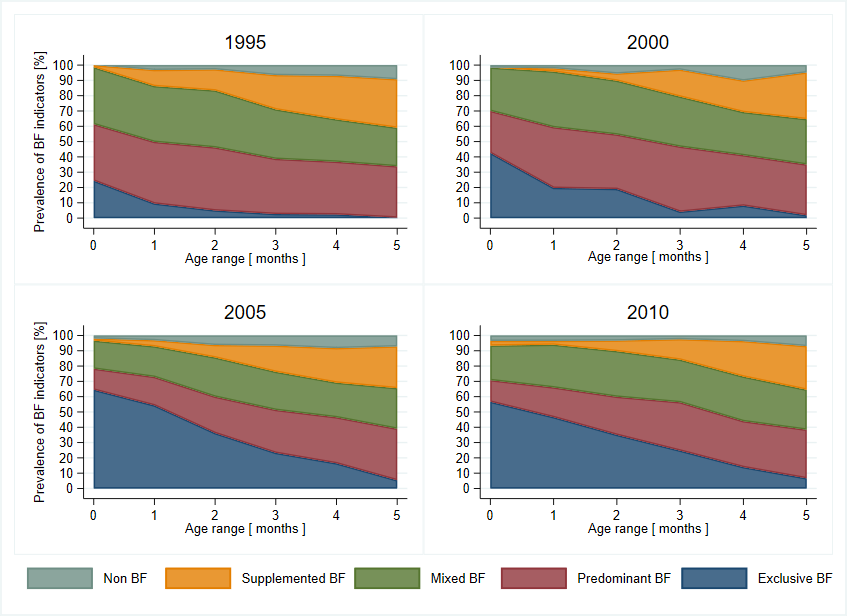
**

**Figure S9.** Prevalence of breastfeeding indicators for infants under six months of age from Colombia by survey year and montly age group. DHS, 1995-2010.
