# Supplementary material for: Evolution of breastfeeding indicators and early introduction of foods in Latin American and Caribbean countries in the decades of 1990, 2000 and 2010
Source: Int Breastfeed J. 2022 Apr 22;17:32. doi: 10.1186/s13006-022-00477-6 (PMC9034574; doi:10.1186/s13006-022-00477-6)
Supplement: Supplementary file 13 — Additional file 13: Figure S11. Prevalence of breastfeeding indicators for infants under six months of age from Guatemala by survey year and monthly age group DHS, 1995–2015. [file 13006_2022_477_MOESM13_ESM.docx]

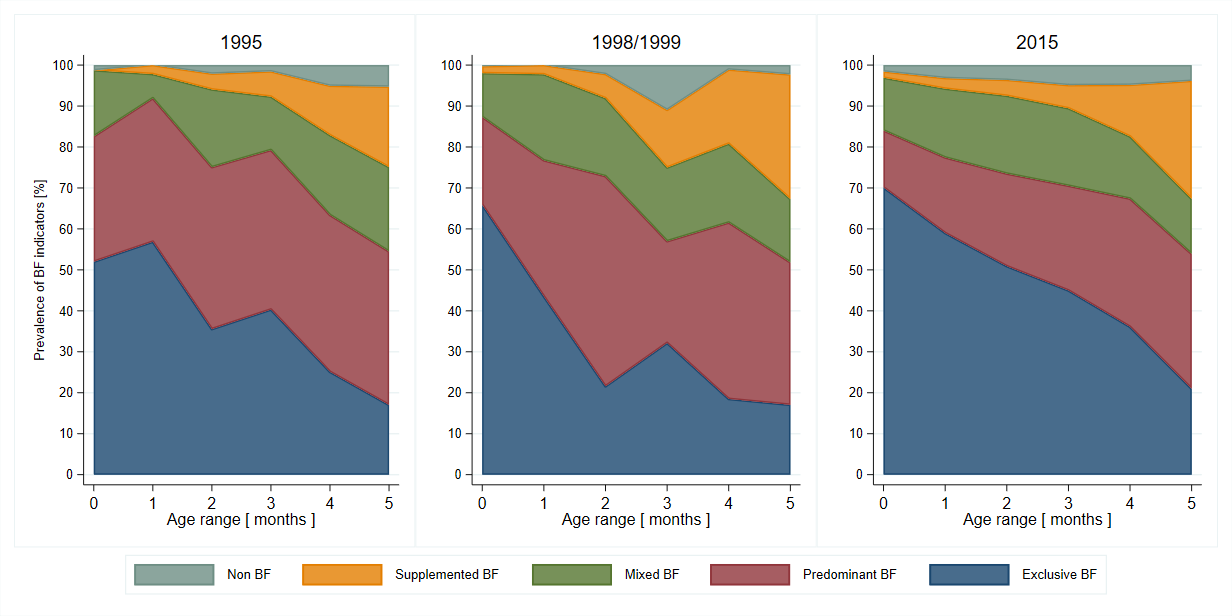


**Figure S11.** Prevalence of breastfeeding indicators for infants under six months of age from Guatemala by survey year and montly age group. DHS, 1995-2015.
