# Supplementary material for: Evolution of breastfeeding indicators and early introduction of foods in Latin American and Caribbean countries in the decades of 1990, 2000 and 2010
Source: Int Breastfeed J. 2022 Apr 22;17:32. doi: 10.1186/s13006-022-00477-6 (PMC9034574; doi:10.1186/s13006-022-00477-6)
Supplement: Supplementary file 14 — Additional file 14: Figure S12. Prevalence of breastfeeding indicators for infants under six months of age from Haiti by survey year and monthly age group. DHS, 1994–2017. [file 13006_2022_477_MOESM14_ESM.docx]

**
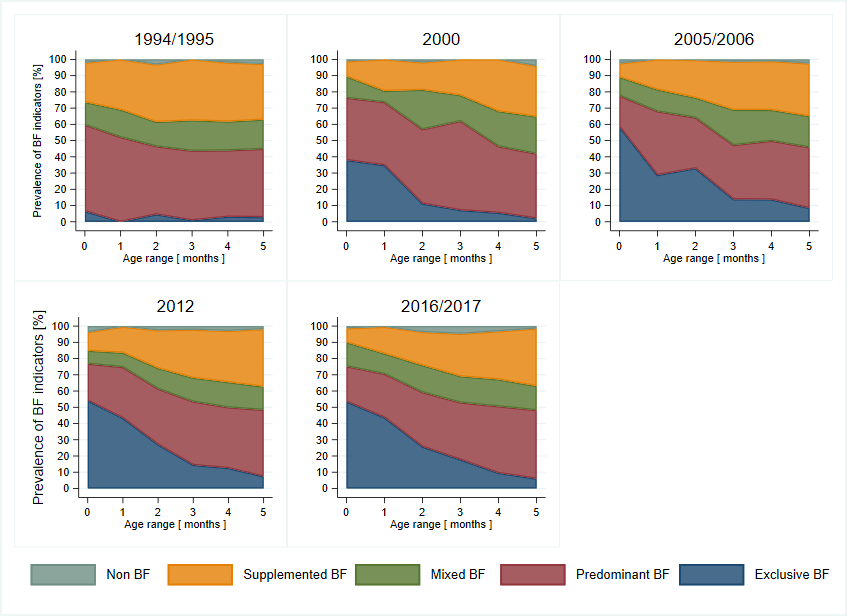
**

**Figure S12.** Prevalence of breastfeeding indicators for infants under six months of age from Haiti by survey year and montly age group. DHS, 1994-2017.
